# Bilateral index, power, force, and velocity during bench press with different loads in male handball players

**DOI:** 10.3389/fphys.2023.1130914

**Published:** 2023-03-22

**Authors:** Andrés González-Ramírez, Carol Torres, Carlos Magallanes, Carlos Gabriel Fábrica

**Affiliations:** ^1^ Institute of Physical Education, University of the Republic, Montevideo, Uruguay; ^2^ Department of Biophysics, Faculty of Medicine, University of the Republic, Montevideo, Uruguay

**Keywords:** bilateral deficit, bilateral facilitation, power output, upper limbs, symmetry indexes

## Abstract

Bilateral index for upper limbs was determined for maximal force, speed and power in 18 male handball players. Variables were individually assessed with a functional electromechanical dynamometer during unilateral and bilateral bench press push-off for 40%, 60%, and 75% of the maximal isometric force. Limb dominance (symmetry indices) and load effects in the bilateral index were analysed. Bilateral index showed a bilateral deficit for power (range = −8.50 to −41.48) and velocity (range = −11.15 to −38.41), that increases with the load (*p* < 0.05). For maximum force, a bilateral facilitation (range = 2.26–5.57), which did not vary significantly as a function of load, was observed. Symmetry indices showed no association with the bilateral index (40% load: r = 0.45, 60% load: r = 0.05, 75% load: r = 0.39). These results contribute to understanding the phenomenon; however, individual-to-individual observation reflects that caution should be kept when assessing an individual athlete. In conclusion, bilateral deficit or facilitation for bench press depends on the variable considered, whereas its magnitude depends on the load. Moreover, limb dominance does not affect it. This finding must be regarded as a general trend, but a different situation may occur during the assessment of a particular athlete.

## Introduction

Bilateral deficit (BLD) is the phenomenon whereby the force produced by both limbs is lower than the sum of the forces produced by the left and right limbs separately (Henry and Smith, 1961; Janzen et al., 2006). The opposite phenomenon is called bilateral facilitation (BLF) (Henry and Smith, 1961). It has been suggested that BLD is caused by neural inhibition when two homologous contralateral limbs attempt to contract simultaneously (Janzen et al., 2006). Other factors such as the exercise involved, level of training, age, predominant muscle fibre type, right-left dominance, and sport practised may also contribute to BLD (Jakobi and Cafarelli, 1998). In athletes such as rowers, cyclists, and weightlifters who use mostly bilateral movements in their training, BLF may even occur (Howard and Enoka, 1991; Skarabot et al., 2016), suggesting familiarity with the task and specificity of the exercise may contribute to reducing the effects of BLD. BLD and BLF are not consistent phenomena, with high variability in magnitude (Howard and Enoka, 1991; Skarabot et al., 2016). BLD and BLF are commonly determined by the bilateral index (BI; BI(%) = [100 x bilateral force/(right unilateral force + left unilateral force)]—100) (Howard and Enoka, 1991). Positive BI is indicative of BLF, while negative BI indicates BLD. BI is usually calculated with force values (Skarabot et al., 2016). Given that, in most sports maximal power is more relevant for performance than maximal force production (Dickin et al., 2011), power may be a more appropriate variable than force to determine BI.

Skarabot et al. (2016) suggest that BLD is more consistent in dynamic contractions, with the magnitude being greater in the lower body than in the upper body movements. However, this conclusion was based on studies conducted only with force records. In addition, from our knowledge, determinations BI during upper body dynamic exercises commonly used in training, such as the bench press, have only one antecedent (Vandervoort et al., 1987). Indeed, this study was carried out with force records and in isokinetic conditions (constant velocity). This aspect is relevant because performance in dynamic movements is related to the ability of muscles to produce high power (product of force and velocity) (Samozino et al., 2010; Ferraro and Fábrica, 2017).

In the last years, some studies have taken into account power for BI estimates, but all of them were carried out considering actions with lower limbs (Pain, 2014; Rejc et al., 2015; Ascenzi et al., 2020; Kozinc and Šarabon, 2021; Nicholson and Masini, 2021; Pleša et al., 2022). Power BI on the bench press deserves to be studied because i) the effect of limb dominance seems to be more prevalent in the upper than the lower body (Leung et al., 2021), ii) the ability to generate high power values is decisive in many sports (Cormie et al., 2011), and iii) bench press is one of the exercises most used both in training and testing (Castillo et al., 2012; Sreckovic et al., 2015; García-Ramos et al., 2016). Power is a variable that arises from the product of force and velocity (Winter et al., 2016), and can be influenced by other factors, for example, coordination (Ferraro and Fábrica, 2017). In line with this, if power BLD or BLF is observed, it could be due to BLD, or BLF, in force, velocity or both. The contribution of BLD and BLF could also vary depending on the load used (Cormie et al., 2011). In this way, the analysis of the BI considering power and its component variables, and analysing the effects of dominance and load in bench press, could broaden our knowledge regarding the factors that determine performance in exercises involving upper limbs.

The main aim of this work is the analysis of the BI for power, force and speed in the bench press in handball players, focused on limb dominance and load effects. Based on previous studies where some these variables were analysed for lower limbs, it was hypothesized that: 1) different loads can result in changes of BI power values; 2) these changes are associated with BI velocity values rather than with BI force values; 3) limb dominance is a relevant factor in explaining the results of BI. To test these hypotheses, loads controlled with a validated functional electromechanical dynamometer (FEMD) (Chamorro et al., 2017; Cerda Vega et al., 2018) were utilized. To minimise factors that may confuse the analyses, a) athletes who practise a sport (handball) in which asymmetric movements with the upper body predominates were selected, b) these athletes were familiarised with the task (bench press) used in the study, c) the similarity of technique between unilateral and bilateral situations was maximised by stabilising the posture position and, therefore, limiting counterbalance compensatory movements. Finally, given that power BI could be potentially useful for performance assessment, sought to determine whether the effect obtained at the population level could be extrapolated to individual assessment.

## Materials and methods

### Participants

The sample size for this work was estimated based on data reported in previous studies (Samozino et al., 2014), and on Cohen’s guidelines (Cohen, 1988), with an alpha level of 0.05 and power level of 0.8. Eighteen male handball players (age 22.1 ± 3.8 years, body mass 84.5 ± 15.9 kg, body height 179.3 ± 7.6 cm, IMC 26.2 ± 4.1) participated in the study. All players were free from injuries, had more than 5 years of sports experience, trained three or more times a week and had athletic proficiency.

Participants were asked to refrain, caffeine, social drugs and alcohol consumption and perform strenuous physical activity 48 hs before the test. All players were fully informed of experimental procedures and possible discomforts associated with the study before giving their written informed consent to participate. The study conformed to the Helsinki Declaration and was approved by the Institutional Research Ethics Committee of ISEF–Universidad de la República (Res. N°13/2022).

### Experimental procedures and variables determination

All tests were carried out during the beginning of the pre-competitive period. Test were carried out throughout 5 days of the same week, in the morning (9–12 h), with a small group of participants (3 or 4) in each day. This was coordinated with their coach so as to not affect their training schedule. Temperature (21.4°C ± 4.3°C), humidity (70.4% ± 6.4%) and atmospheric pressure (1.014 ± 0.004 mbar) were measured before starting each trial. Participants performed a 15-min standardized warm-up and familiarization movements with low loads. They laid supine with their legs crossed above the bench and had to keep their backs on the bench to limit the lower body influence. Subsequently, they performed the tests using a functional electromechanical dynamometer (Dynasystem® Model DynaBlackbox) (DEMF) in tonic mode (Chamorro et al., 2017; Cerda Vega et al., 2018) connected through not extendable ropes to two solid grips for pulley machine.

Participants first performed a maximal isometric contraction with the elbows at 150° of extension (Vandervoort et al., 1987) of the bench press for 5 s in the bilateral condition (BIL), unilateral with the dominant upper body side (ULd), and unilateral with non-dominant upper body side (ULn). Three loads (40%, 60%, and 75%) were determined for each condition based on the maximum force isometric values. Later, each participant performed three bench press repetitions against the three loads established for each condition (BIL, ULn, ULd) in randomized order. In each trial, they were asked to extend their upper limbs as fast as possible. The execution technique of the movement was controlled in an observational way. The rest time between each trial within each load condition was 2 min, and a 5-min break was taken between each condition.

Instantaneous force, velocity and power output during the bench press push were registered directly with DEMF. Peak values of power, force and velocity (Pmax, Fmax and Vmax) were the variables for analysis. The maximum values were used to determine: limb symmetry indices (LSI) (Riemann et al., 2018); and BLD or BLF through BI. Given that, in addition to the load lifted, should overcome approximately 10% of their body mass according to Dempster (1955), this load was considered for force and power computations.

LSI were calculated for each load (40%, 60%, and 75%) according to the relationship:

$$LSI=\frac{value\;in\;ULd}{value\;in\;ULn}\;x\;100$$

Asymmetry was considered when the LSI values were outside the 90%–110% range.

The BLD or BLF was estimated for each load by calculating BI according to the relationship:

$$BI\%=100\;x\;\left[\frac{BIL}{\left(ULd+ULn\right)}\right]-100$$

where the values of Pmax, Fmax, and Vmax obtained in each condition for each load were substituted. A positive bilateral index (BI > 0%) was considered indicative of BLF, while a negative value (BI < 0%) was considered BLD.

Each determination was made individually for each participant. As detailed in the following section, all the analyses were carried out using mean values. However, in the discussion, we considered it would be interesting to present also the values obtained individually; therefore, for some variables, individual results are shown as well.

### Statistical analyses

The Coefficient of Variability (CV), the intra-class correlation coefficient (ICC), and their corresponding 95% confidence intervals were calculated. Acceptable reliability was determined as an ICC >0.70 and CV >15% (Haff et al., 2015). For analyses, we selected the execution in which the higher Pmax was developed in each load condition. Data are presented as mean and standard deviations (SD). The normal distribution of the data (Shapiro-Wilk test) and the homogeneity of variances (Levene test) were confirmed. Differences between load conditions for LSI and BI were analysed with one-way ANOVA for repeated measures and Bonferroni *post hoc* corrections. Pearson correlation coefficients were calculated to examine the relationships between Pmax and the variables Fmax and Vmax and for LSI and BI for each load condition. All statistical analyses were conducted using the free statistical package JASP 0.16.2 with *p* < 0.05. The force of the correlations was interpreted according to Hopkins et al. (2009).

## Results

The CV% and ICC for all the variables considered in this study were 5.6%, 7.3%, and 12.2% for Fmax, Vmax and Pmax, respectively. The ICC were 0.99 [0.98–1,00], 0.89 [0.74–0.97] and 0.76 [0.50–0.92] for Fmax, Vmax and Pmax respectively.

The recorded values of maximum isometric force, from which the percentages were determined, were the following: 314.4 ± 80.9 N, 310.6 ± 79.8 N, and 668.2 ± 172.5 N for ULn, ULd and BIL, respectively. Table 1 shows the mean values of the mechanical variables considered for each load condition. For all loads and conditions, the correlation analyses between Pmax and Vmax were very strong (range r = 0.70 to r = 0.84). The force of the correlations between Pmax and Fmax was more variable with the load and condition. For ULn and ULd conditions with loads of 40% and 75%, correlations were strong (range r = 0.53 to r = 0.66), while for 60% of load, correlations were very strong (range r = 0.70 to r = 0.76). On the other hand, in BI condition for 40% it was near-perfect (r = 0.90), for 60% very strong (r = 0.70) and for 75% strong (r = 0.55).

**TABLE 1:** Values of the variables analysed and results of comparison between the different load conditions.

Variable	40%	60%	75%
Fmax unilateral non-dominat [N.kg^−1^]	23.7 ± 5.1	28.0 ± 5.5	33.8 ± 8.6
Fmax unilateral dominat [N.kg^−1^]	24.4 ± 5.2	28.7 ± 5.3	34.0 ± 6.9
Fmax bilateral [N.kg^−1^]	48.7 ± 10.0	60.0 ± 13.5	69.5 ± 14.3
Vmax unilateral non-dominat [m. s^−1^]	1.87 ± 0.36	1.36 ± 0.41	1.08 ± 0.42
Vmax unilateral dominat [m.s^−1^]	1.81 ± 0.39	1.29 ± 0.39	0.99 ± 0.34
Vmax bilateral [m. s^−1^]	1.65 ± 1.19	0.95 ± 0.20	0.54 ± 0.16
Pmax unilateral non-dominat [W.kg^−1^]	33.3 ± 9.8	31.4 ± 10.2	29.5 ± 10.8
Pmax unilateral dominat [W.kg^−1^]	33.1 ± 11.0	30.6 ± 9.5	28.1 ± 8.7
Pmax bilateral [W.kg^−1^]	57.8 ± 15.6	47.6 ± 11.8	32.4 ± 10.3

The mean and SD, were calculated over 18 values (1 for each participant), each of which was the trial in which maximum power was developed. The normalization of force values was carried out considering that 10% of body mass corresponding to upper limbs.

The average LSI values obtained are presented in Table 2. At the individual level, important asymmetries were observed in a large proportion of the sample. For LSI with Pmax at 40%, fourteen participants; for Pmax 60%, nine participants; and for Pmax 75%, seventeen participants. For LSI with Fmax at 40%, ten participants; for Fmax 60%, five participants; and for Fmax 75%, seven participants. Finally, LSI with Vmax at 40%, nine participants; for Vmax 60%, ten participants; and sixteen participants for Vmax 75%.

**TABLE 2:** Values of LSI for the mechanical variables analysed and results of comparison between the different load conditions.

LSI (%)	40%	60%	75%
Fmax	104.1 ± 15.6	103.3 ± 10.6	102.9 ± 15.8
Vmax	99.8 ± 27.1	96.9 ± 21.3	95.8 ± 26.8
Pmax	104.1 ± 41.5	99.3 ± 18.4	99.6 ± 24.8

The mean and SD, were calculated over 18 values (1 for each participant), each of which was the trial in which maximum power was developed. The LSI, calculated for the different variables did not present significant differences between loads. For comparisons, ANOVA, was performed in the case of Fmax and Friedman’s test for Vmax and Pmax.

The results and analyses of BI are presented in Table 3. As in the LSI analysis, in BI, the individual-to-individual observation showed that for all the variables considered, some participants exhibited BLD and others BLF. For BI with Pmax at 40%, twelve participants exhibited BLD and six participants BLF, for Pmax 60%, fourteen participants exhibited BLD and four participants BLF, and for Pmax 75%, the eighteen participants exhibited BLD. For BI with Fmax at 40%, ten participants exhibited BLD and eight participants BLF, for Fmax 60%, eight participants exhibited BLD and ten participants BLF, and for Fmax 75%, eight participants exhibited BLD and ten participants BLF. Finally, BI with Vmax at 40%, fourteen participants exhibited BLD and four participants BLF, for Vmax 60%, fifteen participants exhibited BLD and three participants BLF, and for Vmax 75%, seventeen participants exhibited BLD and one participant BLF.

**TABLE 3:** Values of BI for the mechanical variables analysed and results of comparison between the different load conditions.

BI (%)	40%	60%	75%
Fmax	2.26 ± 16.81	5.77 ± 15.37	3.52 ± 17.27
Vmax *	−8.50 ± 15.34	−23.56 ± 25.49	−41.48 ± 24.91
Pmax*	−11.15 ± 19.18	−19.24 ± 24.21	−38.41 ± 24.41

The mean and SD, were calculated over 18 values (1 for each participant), each of which was the trial in which maximum power was developed. The (*) indicates the variables for which BI, presented significant differences between load conditions. Negative signs in the mean BI, values indicate BLD, while positive signs indicate BLF. The *post hoc* shows differences for speed between 40% and 60% (*p* < 0.05); and 40% and 75% (*p* < 0.001); and 60% and 75% (*p* < 0.05) and for power between 40% and 75% (*p* < 0.01); and 60% and 75% (*p* < 0.05).

Correlation between LSI and BI were weak or moderate and not significant: at 40% load: r = 0.45, *p* = 0.06; at 60% load: r = 0.05, *p* = 0.85; and at 75% load: r = 0.39, *p* = 0.11.

## Discussion

This study was designed to explore BI during bench press with different loads, considering power as the variable of primary interest. As power is a composite variable (Samozino et al., 2010; Winter et al., 2016), force and velocity were also considered and used to calculate BI. Two aspects were analysed: the effects of limb dominance and the effects of load.

In all the situations analysed, Pmax was very strongly correlated with Vmax, and the correlation with Fmax, although it was at least strong, does not remain constant when changing the load. This finding is consistent with previous work and confirms that in the bench press, as in other fast movements used in testing and training, power depends on velocity rather than force (Ferraro and Fábrica, 2017).

The averaged results of LSI calculated with Pmax, Fmax, and Vmax were within the range of 90%–110%, and they did not change with the load. According to Riemann et al. (2018), these values indicate no asymmetry in the capacity to develop maximum values of the three variables in any load conditions studied. However, two facts must be highlighted: firstly, a large variability was observed, particularly for the calculations made with Pmax and Vmax; secondly, individual-to-individual observation reflects that most participants exhibited asymmetries. In fact, except in Fmax for some load conditions, more than 50% of the participants were outside the range that allows symmetry to be assumed. This result is different from the ones obtained in studies using other exercises (vertical jumps, for example,); thus, it may be relevant to conduct future research comparing the bench press with other exercises. Related to this, Samozino et al. (2014) used two force platforms which allowed them to measure each member separately during a bilateral action and determine the asymmetry with these data. An experimental design that allows a similar approach for bench press could allow a deeper discussion of the relevance of asymmetries in this exercise. Our findings are based on determinations during unilateral actions; thus, they are only an approximation and allow us only to suggest that asymmetry, in general terms, does not explain the values of BI but should be considered when evaluating a particular subject during the bench press.

Our results for BI showed that BLD and BLF depend on the variable considered, whereas their magnitude depends on the load used in the exercise. For Pmax, our primary variable of interest, BLD was observed, and it increased with the load; the same was found for Vmax; while with Fmax, BLF was observed, and these did not vary significantly with the load. Caution should be taken when comparing our results to those obtained in other studies because the exercise and the variable consider most relevant differ from those of the antecedents. Besides that, not all studies randomised the unilateral and bilateral conditions. Moreover, fatigue and potentiation might also have affected the result of BI in previous studies (Jakobi and Chilibeck, 2001). Even so, some considerable differences with previous studies deserve to be analysed.

Considering that the instruction was to perform the push as fast as possible and that we used the maximum values for the BI determinations, we expected that the results would be similar to those reported for fast movements with lower limbs. However, Samozino et al. (2014) found for maximum force a BLD of 36.7% ± 5.7%, a value very far from the BLF range found in our work for that variable (2.26 ± 16.81 to 5.77 ± 15.37). This difference agrees with the finding that upper-body movements generally show lower BI than lower-body movements (Skarabot et al., 2016). Beyond that, the most remarkable result of our study was the enormous variability when comparing it with others. The BI calculated with Pmax in our work showed values close to those found in studies for lower limbs, particularly with high loads, and the same happened with BI determined from Vmax. Based on these results, some interpretations emerge that should be considered in future studies using the bench press. First, when evaluating the existence of BLD or BLF, the calculation should be made with a variable that is consistent with the primary objective of the exercise; a determination made with Fmax may lead to a different result than that obtained with Pmax or Vmax. Second, the determination of BLF or BLD with Pmax or Vmax follows the same trend. Finally, the magnitude of the BI depends significantly on the load used in the exercise.

Regarding our BI results, it is also worth clarifying, as we did when discussing LSI, that the calculations were not made with the individual values during the bilateral action, as in Samozino et al. (2014), for example,. This type of determination, which was not possible in our work, would allow us to discuss the effect of potential differences in postural stabilisation (Herbert and Gandevia, 1996). Although we control the posture during the exercise, changes in muscle actions that we cannot control may affect torque production during unilateral conditions and thus affect BI values (Simoneau-Buessinger et al., 2015).

In summary, despite the large variability observed for BI values in the bench press, when it is determined with Pmax or Vmax, most participants tend to exhibit BLD. Since the premise was to make the gesture as fast as possible, our results suggest that there is BLD in the bench press, which manifests more clearly with high loads. Based on what we have discussed, we concluded that our first two working hypotheses were fulfilled. Finally, it is important to remark that these results cannot necessarily be extrapolated to other populations of athletes. This population of handball players was selected so as to minimize the appearance of factors that may affect the analysis of the proposed objectives. However, future studies with other populations are necessary in order to discuss in greater depth the effects of the use of power and different variables for BI calculus.

## Data Availability

The raw data supporting the conclusion of this article will be made available by the authors, without undue reservation.
